# Effect of the Refining Process on Total Hydroxytyrosol, Tyrosol, and Tocopherol Contents of Olive Oil

**DOI:** 10.3390/foods9030292

**Published:** 2020-03-05

**Authors:** Paolo Lucci, Valentina Bertoz, Deborah Pacetti, Sabrina Moret, Lanfranco Conte

**Affiliations:** 1 Department of Agri-Food, Animal and Environmental Sciences, University of Udine, via Sondrio 2/a, 33100 Udine, Italy; bertoz.valentina@spes.uniud.it (V.B.); sabrina.moret@uniud.it (S.M.); lanfranco.conte@uniud.it (L.C.); 2 Department of Agricultural, Food, and Environmental Sciences, Marche Polytechnic University, Via Brecce Bianche, 60131 Ancona, Italy; d.pacetti@staff.univpm.it

**Keywords:** hydroxytyrosol, lampante oil, olive oil, phenolic compounds, refining, tyrosol, tocopherols, ultra-high performance liquid chromatography

## Abstract

The impact of the olive oil refining process on major antioxidant compound levels was evaluated by means of UHPLC analysis of lampante olive oils collected at different stages of the refining procedure (degumming, chemical and physical flash neutralization, bleaching, and deodorization). For this purpose, the evolution of the tocopherol fraction was investigated by means of the UHPLC-FL method, while the influence of the refining process on the total hydrolyzed phenolic content was assessed by measuring hydroxytyrosol and tyrosol levels after acid hydrolysis of the phenolic extracts. Refining was found to have a marked effect on total hydroxytyrosol and tyrosol contents, as they are completely removed in the early steps of the refining procedure. In contrast, the variation trends of tocopherols are not always clear-cut, and significant decreases in content from 7% to 16% were only revealed during refining in four out of nine samples. In addition, five of the nine refined oils showed final tocopherol concentrations higher than 200 mg/kg, the limit imposed by international standards regarding the content of such compounds in commercial olive oils. This study supports the need for a revision of the International Olive Oil Council (IOC) standard relative to the limit established for tocopherol addition to refined oils to avoid possible legal and economic trade issues.

## 1. Introduction

Extra virgin and virgin olive oils are obtained solely through physical means by mechanical or direct pressing of the olives. They are not subjected to any treatment other than washing, decantation, centrifugation, and filtration, and they may be consumed without refining. In contrast, “lampante” olive oils, which represent another category of mechanically extracted olive oils, are unfit for direct human consumption and have to be refined prior to use. According to the International Olive Oil Council (IOOC), refined olive oil is the olive oil obtained from lampante olive oils by refining methods that do not lead to alterations in the initial glyceridic structure. It has a free acidity, expressed as oleic acid, of no more than 0.3 g oleic acid/100 g oil, and its other characteristics correspond to those fixed for this category in the IOOC standards [1]. 

Most of the world’s olive oil is produced in countries in the Mediterranean Basin. However, a significant amount of oil produced in the Mediterranean area (over 20% of total production) is of such poor quality that it must be refined in order to be fit for human consumption [2]. One main purpose of a refining plant is to produce edible oils with low acidity values [3]. Moreover, other minor substances that can affect the quality of the oil, such as phospholipids, pigments, peroxides, traces of metals, herbicides, and volatiles responsible for sensory defects, are also removed or their content reduced during refining treatments [4,5]. It should be stressed, however, that although refining aims to extend the shelf-life of oils by removing undesirable compounds, it also results in the loss of molecules of biological and technological interest such as tocopherols and phenolic compounds [6]. Polyphenols present in olive oil act as antioxidants mainly in the initial phase of autoxidation by scavenging free radicals and as chelating agents against metals [7,8]. The phenolic compounds present in olive oil include tyrosol, hydroxytyrosol, hydroxybenzoic acid, oleuropein, caffeic acid, vanillic acid, *p*-coumaric acid, and tyrosol and hydroxytyrosol derivatives [9]. On the other hand, tocopherols, which are recognized as important lipid oxidation inhibitors in food and biological systems, represent the most abundant lipophilic antioxidants in olive oils, with levels usually ranging from 5 to 400 mg/kg, with α-tocopherol being the main isomer [5,10,11]. 

The quantity of antioxidants that are lost during the refining process depends on the severity and the conditions employed. In the neutralization step, phenolic substances are destroyed or reduced. Tocopherols can also be partially removed during the deodorization/distillation steps in both chemical and physical refining processes [12]. For instance, in physical neutralization, tocopherols may be distilled and removed from sample together with small amounts of sterols, about 100% of squalene, etc. [6,13]. The decrease in antioxidant levels as a result of the refining process is relevant because oxidative stability is not only a quality parameter but also represents one of the most important oil indicators of shelf-life [14]. During oxidation, compounds responsible for unpleasant odors are produced, thus making the oil less acceptable or unacceptable for the consumer or for the industries that use it as an ingredient. In edible oils, oxidation is influenced by energy sources such as light or heat, concentration and type of oxygen, oil treatment, minor components such as transition metals and pigments, and antioxidant content, among others [15]. For this reason, the supplementation of tocopherols is allowed both by the Codex Alimentarius and by the IOC for refined oils only, with the aim of restoring natural tocopherols lost in the refining process [1,16]. However, the concentration of α -tocopherol in the final product must not exceed 200 mg/kg, thus representing a problem in international trade in some cases because “high-quality” olive oils (mixtures of refined and extra virgin olive oils) can frequently and naturally exceed the set limit. Therefore, this study aimed (1) to examine the effect the different refining steps would have on the tocopherol content of lampante oil samples; and (2) to determine whether the magnitude of their content decrease would have practical implications regarding the maximum level established for α-tocopherol by the international standards for olive oils. Additionally, considering the impact of polyphenols on oil stability as well as the health claims recently approved by the European Food Safety Authority for olive oil polyphenols (Commission Regulation (EU) 432/2012) [17], (3) the influence of the refining processes on the total content of hydroxytyrosol and tyrosol after acid hydrolysis was also investigated by applying a UHPLC-UV method.

## 2. Materials and Methods 

### 2.1. Chemicals

Acetone, acetonitrile, isopropanol, ethanol, methanol, and *n*-hexane, (all HPLC grade) were purchased from Sigma–Aldrich (Milano, Italia). Water was purified with a Milli-Q system (Millipore, Bedford, MA, USA). All other reagents were of analytical grade. 5α-cholestanol, tocopherol (α, γ and δ-tocopherols), and phenolic acid (tyrosol and hydroxytyrosol) standards were purchased from Sigma–Aldrich (Milano, Italia). 

### 2.2. Industrial Samples

Lampante olive oil samples (*n* = 9) were industrially refined using the common refining process under the following conditions: degumming with deionized water for 30 s at 80 °C; chemical neutralization up to about 0.2% of free acidity with sodium hydroxide (23.5%); physical flash neutralization at 230 °C at 1 mbar to reach about 0.02% free acidity; bleaching with 3% bleaching earth mix containing 5% activated carbon at 90–97 °C at 20 mbar; and deodorization at 200 °C at 2 mbar for about 2.5 h. Oil samples (3 L) were therefore taken at different stages of the refining procedure (crude oil, degummed oil, neutralized oil, bleached oil, deodorized oil) and kept frozen in dark glass bottles at −18 °C until chemical analyses were performed.

### 2.3. Commercial Samples

Eleven commercial refined olive oils were purchased at local markets.

### 2.4. Analytical Determinations for Quality and Purity Assessment

Determination of acidity as oleic acid and specific UV absorption at 232 nm (K232) and 270 nm (K270), as well as quantification of fatty acid methyl esters, sterols, dialcohols, and waxes, were performed according to the procedure reported in Regulation EC/2568/91 [18]. The difference between theoretical and experimental equivalent carbon number 42 (ΔECN42) was determined according to the IOC method for ECN42 analysis [19].

### 2.5. UHPLC Analysis of Tocopherols

For the determination of tocopherols in oil samples, a new UHPLC method was developed and validated in-house. Analysis were realized using a Shimadzu Nexera (Shimadzu, Kyoto, Japan) UHPLC coupled with same components used for polyphenols analysis and the fluorescence detector RF-20Axs with double acquisition channels and a 12-µL cell. The detector wavelengths were set at 296 nm (excitation energy) and 325 nm (emission energy). Acquisition frequency was 10 Hz. The sample was diluted in 2-propanol at a 100 mg/mL concentration and 1 μL injected on column as compromise between the required sensitivity and the capacity of the column. The chromatographic separation was performed using an Agilent Eclipse PAH column (1.8 µm particle size, 4.6 mm × 50 mm) under isocratic elution using as a mobile phase a mixture of methanol/acetonitrile (60/40, *v*/*v*) with a flow rate of 600 μL min^−1^. Oven temperature was set at 30 °C. The injected volume for each sample was 1 μL. Tocopherols were quantified using three different calibration curves for α, γ, and δ in the range 0.05–100 ng injected on column. The performance of the UHPLC method was deeply investigated (linearity, repeatability, matrix effect) in order to check its suitability for tocopherol detection in oil samples.

### 2.6. UHPLC Analysis of Phenolic Compounds

Extraction of phenolic compounds was performed according to the official IOC method [20]. Then, acid hydrolysis was applied following the procedure suggested by Rovellini et al. [21]. Briefly, 1 mL of methanolic extract was dried on a warm bath at 40 °C under a gentle steam of nitrogen and then hydrolyzed with 1 mL of ethanol/water/sulfuric acid (50/40/20, *v*/*v*) at 40 °C for 1 hour. The sample was stored in the dark at room temperature for 12 hours before injection in UHPLC. The hydrolyzed phenolic compounds were analyzed using an Agilent Poroshell 120 EC-C18 reversed phase column (2.7 µm particle size, 4.6 × 150 mm) on a Shimadzu Nexera UHPLC System (Shimadzu Nexera, Kioto, Japan) equipped with dual pump LC-30AD, on-line degasser DGU-20AS, column oven CTO-30A, auto sampler SIL-30AC, and diode array detector (SPD-M20A). Gradient separation was created from solvent A (2% acetic acid in water) and solvent B (acetonitrile) as follows: 0–1min, isocratic condition at 95% A; 1–12min linear gradient from 5 to 70% B; 12–13 min, linear gradient from 70 to 90% B; isocratic condition kept up to 17 min; 17 min back to initial condition at 95% A; isocratic step kept up to 22 min for column conditioning. The mobile phase flow rate was 450 μL min^−1^. The column temperature was 30 °C. The injected volume for each sample was 5 μL. The detector was set at 280 nm. Polyphenol quantification was obtained through calibration curves in the range of 10–600 ng of tyrosol and hydroxytyrosol injected on column with R^2^ values higher than 0.999, in all cases.

### 2.7. Statistical Analysis

All experiments were carried out in triplicate (with the exception of measurements on industrial samples reported in Table 1 and Table 2). Data were expressed as means ± standard deviation (SD). Differences of the analyzed compounds (tocopherols, polyphenols) among samples at different refining steps were calculated using one-way analysis of variance (ANOVA) with Tukey’s post hoc procedure, with a level of significance at *p* ≤ 0.05 (R Project for Statistical Computing; R Foundation for Statistical Computing, Wien, Austria). Principal component analysis was performed by using software R (R Core Team, 2013).

## 3. Results and Discussions

### 3.1. Characterization of Selected Oil Samples

The chemical characteristics of the analyzed samples are presented in Table 1 and Table 2. Regarding quality parameters, free acidity of crude oils collected for the study ranged from 2.2 to 5.1 % of free oleic acid/100 g oil. Samples 7, 8, and 9 are characterized by a free acidity close to the maximum level established by the IOC trade standard for edible virgin olive oil [1], which in this case also included ordinary virgin oil with the upper free acidity limit of 3.3%. Meanwhile, with respect to EU Legislation, which set a limit for virgin olive oil of 2.0 %, all our samples were classified in the lampante category (Table 1). The specific extinction coefficient at 232, which provides information on the presence of primary oxidation products, classifies, according to the IOC trade standard, samples 2 and 6 as lampante and sample 5 as virgin, while the remaining samples meet the extra virgin olive oil (EVOO) limit. Regarding UV extinction coefficient at 270 nm, samples 1, 3, and 6 lie within the limit fixed for ordinary virgin olive oil and samples 2 and 5 exceed this limit, thus being considered as lampante, while samples 7, 8, and 9 respect the limit value for EVOO. In all cases, levels of Delta-K, a parameter not considered for lampante oils in the EU regulations, were below the maximum level fixed for extra-virgin and virgin olive oils. 

On the other hand, the assessment of purity parameters reveals, as expected, that all samples had been obtained from olives by means of mechanical or other physical processes (Table 2).

The fatty acid (FA) composition (%) of oil samples was studied within the official limits established for olive oil referred to in the IOC trade standard applied to olive oils and olive pomace oils. The results showed a typical olive oil FA profile with oleic acid higher than 66.2% and palmitic acid lower than 15.5% in all cases, among others. At the same time, according to the established limits, the absolute value of the difference between theoretical and experimental ECN42 content (ΔECN42) was always lower than |0.2|. 

Sterol composition (%), which is one of the most suitable indicators for the determination of the botanical origin of oils, was also assessed revealing the absence of adulteration or contamination of olive oil with extraneous vegetable oils and/or refined ones. This scenario is also reinforced by the levels encountered of waxes (≤ 300 mg/kg) and erythrodiol + uvaol (≤ 4.5 % of total sterols). 

### 3.2. Analysis of Polyphenols 

Phenolic compounds have been not only correlated with the oxidative stability of oils, but also with high health benefits. According to Regulation (EU) No 432/2012 (“Olive oil phenolic compounds contribute to the protection of blood lipids from oxidative stress”), health effects can be claimed only if the oil contains more than 5 mg of hydroxytyrosol and its derivatives (e.g., oleuropein complex and tyrosol) in 20 g of oil [17]. The official HPLC method for the determination of biophenols in olive oils (Method COI/T.20/DOC. 28/Rev. 2-2017) is based on direct extraction of the biophenolic minor polar compounds from sample by a methanol/water 80/20 (*v*/*v*) solution and subsequent quantification by HPLC-UV at 280 nm using syringic acid as internal standard [20]. However, this analytical approach allows us to obtain only a rough estimate of the amount of phenolic compounds because it is founded on the assumption that the UV response of all biophenols is similar, so the quantification is based on the ratio between the response factors of two of them. Therefore, although there are previous reports focused on the loss of phenolic compounds during refining of different vegetable oils, this is the first time that phenol content has been assessed by means of the UHPLC-UV technique after acid hydrolysis for obtaining phenolic acids in their free form. The sample treatment procedure, which employs sulfuric acid [21] for the hydrolysis of the phenolic extracts, allows for the proper quantification of hydroxytyrosol and tyrosol as well as the correct evaluation of their evolution during the different refining steps. Moreover, only a few studies have investigated the effect of refining on the olive oil matrix. For instance, Kostadinovic-Velickovska and Mitrev [22] observed a reduction of the total phenolic content from 19.23 to 1.82 mg of gallic acid equivalent/10 g of oil in sunflower oil, while more recent research reported losses of 63% of polyphenolic compounds during neutralization, 16% during bleaching, and 67% during the deodorization step of rapeseed oil [23].

**Figure 1:**
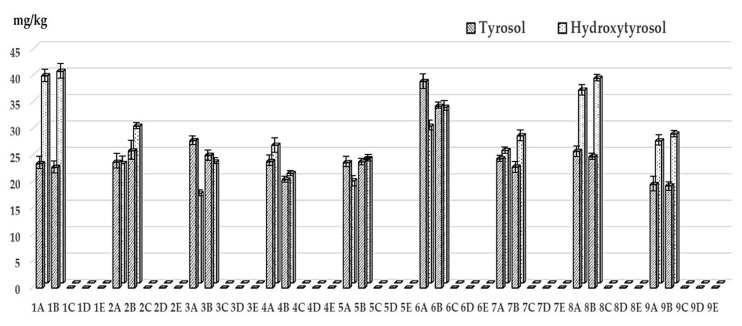
Effect of the refining steps on the hydroxytyrosol and tyrosol content (mg/kg oil). Legend for refining steps: A: Crude oil; B: degummed oil; C: neutralized oil; D: bleached oil; E: deodorized oil.

Our experimental results show that oil samples collected for the study were characterized by initial low contents of total hydroxytyrosol and tyrosol, with values ranging from 43 to 68 mg/kg, thus very far from the fixed level (250 mg/kg) for the health claim on “olive oil polyphenols” (Figure 1). Regarding the evolution of these compounds during refining, a complete removal of polyphenols occurred during the process as they were not detected starting from neutralized samples (Figure 1). Meanwhile, no significant changes were observed between crude and degummed oil samples. This finding is similar to those from a previous study [24]. In fact, *o*-diphenols can be easily oxidized under alkaline conditions, thus explaining their loss during this step [25]. On the contrary, other authors have also reported residual amounts of total polyphenols (0.025–3.2 mg/kg) measured by Folin–Ciocalteau [26,27] in refined olive oils as well as different behaviors between tyrosol and hydroxytyrosol. For instance, Garcia et al. [25], showed that while most *o*-diphenols, including hydroxytyrosol, were lost due to oxidation and polymerization processes occurring under alkaline conditions, tyrosol was accumulated in the soapstocks and was mainly removed after the deodorization phase. Such discrepancies may be however explained by the different conditions that can be chosen for lampante olive oil refining, as well as by the different analytical approaches used for the evaluation of phenolic content (i.e., colorimetric versus chromatographic methods, hydrolyzed versus unhydrolyzed samples, etc.). 

In general, however, our results confirm that polyphenols present in commercial olive oils come mostly from the virgin olive oil blended with refined oil.

### 3.3. Analysis of Tocopherols

For the analysis of tocopherols, a UHPLC method was developed and validated in-house. To improve tocopherol separation, a reversed-phase column packed with a 1.8-µm particle size was employed (Agilent Eclipse PAH column, 4.6 mm × 50 mm) because of the faster separations that can be obtained compared to columns packed with 3- or 5-μm particles [28]. UHPLC separation was performed under isocratic elution (methanol/acetonitrile (60/40, *v*/*v*)), thus eliminating the column re-equilibration step required in gradient separations. The total run time was 5 minutes, with a significant reduction in analysis time and solvent consumption compared to normal-phase IUPAC methodology for tocopherol analysis. Additionally, the reduction in solvent consumption (3 mL per sample) also ensures compliance with the Registration, Evaluation, Authorisation and Restriction of Chemicals (REACH) directive (EC Reg. 1907/2006) [29], aimed at reducing, among other aspects, the environmental impact of the use of chemical reagents in laboratories.

For the evaluation of the linearity, a calibration curve for each α-, γ-, and δ-tocopherol was constructed. Different amounts of tocopherols were injected, ranging from 0.5 to 100 ng on the column. A satisfactory linear dependence of the peak areas with analytes injected on the column was found, with a correlation coefficient higher of 0.999 in all cases. The wide linear dynamic range of the proposed methodology permits the analysis of wide-ranging contents of tocopherols in vegetable oils. Precision was determined by measuring the repeatability of peak areas on replicate injections (*n* = 9) with two different levels of tocopherol standards corresponding to 5 and 50 ng injected on the column. The experiment was also conducted by analyzing the tocopherol content in a commercial refined olive oil sample in order to highlight possible matrix effect. The latter is of extreme importance, especially considering that the proposed method minimizes sample preparation to a simple dilution of oil samples with isopropanol. Results were satisfactory, with repeatability relative standard deviation (RSDr) ≤2.5% in all cases, indicating excellent repeatability of the proposed analytical procedure for real oil sample analysis. The instrumental limits of quantification (LOQs) were 480 pg (α-tocopherol), 230 pg (δ-tocopherol), and 280 pg (γ-tocopherol) injected on the column, based on a signal to noise ratio (S/N) = 10:1.

Table 3 shows the level of total tocopherol content (mg/kg) in olive oil samples taken from the degumming, neutralizing, bleaching, and deodorizing steps. 

**Figure 2:**
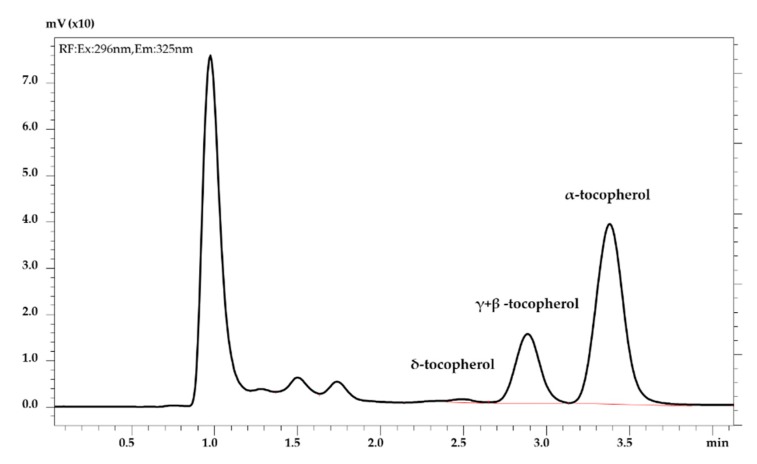
UHPLC-FL trace of tocopherols in lampante olive oil.

Data showed variations in the levels of tocopherols encountered in the nine crude oil samples, with values ranging from 142 to 344 mg/kg. As expected, α-tocopherol was the predominant form in all samples. An example of chromatogram obtained is reported in Figure 2.

As already mentioned for phenolic compounds, for tocopherol only a few studies have evaluated the impact of the refining process on its content in olive oil samples, while most published articles are focused on other types of vegetable oils [30,31,32]. In general, a continuous decrease of tocopherol content has been observed during the refining procedure in sunflower, rapeseed, and soybean oils [6,33]. For instance, a huge decrease in total tocopherol content was observed in soybean oil (45.5%), with a considerable reduction in individual and total tocopherol levels at almost every stage of refining. A gradual but not statistically significant decrease of tocopherol content (14.0%) during the overall chemical refining process has been also reported for sunflower oil [12]. On the other hand, Suliman et al. [34] reported a significant decline of tocopherols, from 750 mg/kg in crude sunflower oil to 520 mg/kg in refined samples. Major decreases have been observed during caustic neutralization because of the reduced stability of tocopherols in the presence of longer contact time with air and alkali [13]. Other researchers revealed a reduction of tocopherol content in olive oil of 37.7% (from 172.5 mg/kg to 107.5 mg/kg) and 23.7% (from 107.5 mg/kg to 82.0 mg/kg) after the degumming–bleaching and steam distillation steps, respectively, with a total loss of 52.5%. In our research, however, the trends were not always clear-cut, and differences among samples behavior were found for different refining steps. Regarding the overall changes observed in tocopherol levels, the maximum decrease of about 16% was revealed for sample 9. Similar reductions were also detected for samples 8, 7, and 2, with final losses of about 11%, 8%, and 7%, respectively. It should be noted, however, that only four out of nine samples showed a statistically significant decrease (*p ≤* 0.05), while in some samples (1, 4, 5, and 6) the refining procedure did not induce any substantial decrease of total tocopherols. Moreover, in sample 3, a significant increase (8.4%) of tocopherols was encountered in the final deodorized sample. Some loss of tocopherols can also occur by evaporation during high temperature deodorization and physical refining, with the magnitude of this decrease depending on the conditions employed. For instance, in soybean oil, after 120 min at 300 °C (a drastic treatment), the tocopherols almost completely disappeared, whereas the reduction during physical refining at 240 °C for 120 min was only about 15%–20% [35]. The latter conditions are closer to those ones employed for the industrial refining of our lampante oil samples where deodorization at 200 °C and 2 mbar for about 2.5 hours was conducted, thereby explaining the reduced effect on total tocopherol contents.

In some cases, this trend could be interpreted as a concentration effect: higher initial free acidity in crude oil results in a greater loss of oil mass which does not necessarily involve the tocol fraction (Table 4). This aspect should be taken into consideration when examining the dynamics that influence the concentration of tocopherols of individual samples during the refining process. Therefore, besides the fact that tocopherols can suffer in the presence of oxygen and the alkaline medium, the small increases in tocols observed in our study for same samples during the degumming/neutralization phase may be associated with an increase in the tocopherol concentrations rather than their absolute values. In fact, as can be seen in Table 4, the loss rate of oil observed during the refining procedure is strongly related to the initial free acidity levels. As a result, a relationship between the initial free acidity of crude oil samples and the extent of decrease of tocopherols during refining could be hypothesized, as crude oil samples characterized by high free acidity resulted, with the exception of sample 2, in refined samples with smaller changes in final tocopherol content. 

**Figure 3:**
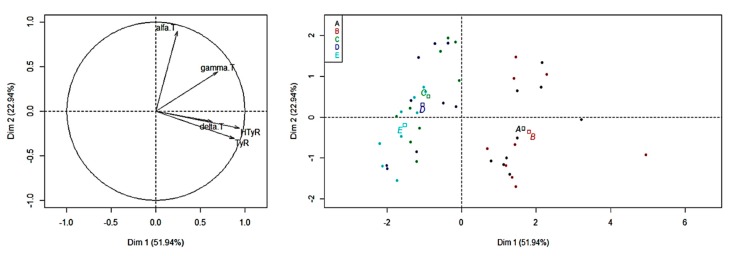
Principal component analysis (PCA) of phenolic compounds and tocols in oil samples across the refining procedure. (**a**) Loading plot of the variables; (**b**) Score plot of the samples. Legend. A: Crude oil; B: degummed oil; C: neutralized oil; D: bleached oil; E: deodorized oil; alfa T: α-tocopherol; gamma T: γ-tocopherol; delta T: δ-tocopherol; HTyR: hydroxytyrosol; TyR: tyrosol.

A general overview of our results can be highlighted by the PCA model reported in Figure 3. The plots show a quite clear homogeneity among samples as it is not possible to distinguish them based on the differences in tocopherol and polyphenol levels detected at different refining steps. The PCA showed only a small grouping effect for bleached and deodorized oil samples. This is probably due to the greater influences of phenolic acids changes occurring after the neutralization process, where they are completely removed from samples. 

In conclusion, our study shows that the refining process has a marked effect on the final content of phenolic compounds, which are totally eliminated at the early stages of the refining procedure, while the effect on the level of tocopherols is minimal and in many cases insignificant (*p* > 0.05). These results also suggest that it is not always necessary to add tocopherols to refined samples with the aim of restoring natural tocols lost in the refining process, as the threshold value fixed by the international standards would be exceeded. In fact, it should be noted that the final concentration of tocopherols is higher than 200 mg/kg in five of nine samples (Table 3). Without any tocols additions, these values do not respect the maximum level imposed by the standards, leading to possible problems in international trade. On the other hand, crude oils characterized by initial tocopherol values lower than 200 mg/kg, showed only a slight decrease in tocopherols in the finished products. These results are also consistent with those shown in Table 5, which reports data obtained by analyzing 11 commercial refined olive oil samples purchased at a local market. As can be seen, 8 out of 11 samples presented a total tocopherol content higher than the maximum allowed value, with 5 samples showing levels even greater than 300 mg/kg.

Finally, this study supports the need for a revision of the IOC standard relative to the limit established for the addition of tocopherols to refined oils and therefore the maximum content allowed for commercial olive oils (mix of refined and virgin olive oils). This is extremely important, especially in the case of “high quality” olive oils which are obtained by mixing refined and extra virgin olive oil. In fact, due to a possible reduced loss of precious antioxidants in the refining phase, legal and economic trade issues could easily arise.

## Figures and Tables

**Table 1:** Quality parameters of selected lampante oils.

Quality Parameters	Samples								
	1	2	3	4	5	6	7	8	9
Free acidity (%)	4.0	5.1	4.0	4.7	5.1	3.7	2.2	2.2	2.7
K232	2.22	2.61	2.30	2.19	2.51	2.89	2.33	2.16	2.25
K270	0.28	0.33	0.28	0.24	0.31	0.28	0.20	0.19	0.20
Delta-K	0.01	0.01	0.01	0.01	0.01	0.01	0.00	0.00	0.00

**Table 2:** Quality parameters of selected lampante oils.

Purity Parameters	Samples								
	1	2	3	4	5	6	7	8	9
∆ECN42	|0.1|	|0.2|	|0.1|	|0.0|	|0.1|	|0.1|	|0.1|	|0.1|	|0.1|
3,5-stigmastadienes (mg/kg)	0.07	0.25	0.44	0.03	0.08	0.04	0.04	0.03	0.06
Cholesterol (%)	0.20	0.28	0.16	0.17	0.22	0.20	0.16	0.14	0.16
Brassicasterol (%)	0.07	0.08	0.11	0.06	0.08	0.06	0.00	0.01	0.02
Campesterol (%)	3.28	3.45	3.41	3.42	3.39	3.29	3.37	3.55	3.52
Stigmasterol (%)	1.45	1.58	1.80	1.94	1.87	1.55	1.61	1.48	1.51
β-sitosterol (%)	94.2	93.6	93.4	93.6	93.5	93.6	93.4	93.9	93.8
∆7-avenasterol (%)	0.40	0.41	0.38	0.38	0.44	0.70	0.44	0.54	0.61
∆7-stigmastenol (%)	0.33	0.35	0.30	0.35	0.39	0.50	1.12	0.35	0.37
Total sterols (mg/kg)	1657	1483	1397	1432	1440	1623	1687	1649	1675
Erythrodiol + uvaol (%)	2.96	2.50	2.41	2.68	1.97	2.32	2.43	2.24	3.04
Waxes (mg/kg)	246	252	261	295	248	246	170	152	207
Aliphatic alcohols (mg/kg)	305	234	255	256	211	322	340	318	380
Fatty acid composition									
C16:0 (%)	11.9	12.6	11.7	12.1	12.4	14.0	15.3	15.8	15.4
C18:0 (%)	3.0	2.6	3.0	2.9	2.8	2.5	2.3	2.3	2.3
C18:1^Δ9c^ (%)	75.4	74.1	76.2	75.7	74.6	69.4	67.6	66.2	66.9
C18:2 ^Δ9c,12c^ (%)	6.9	7.8	6.3	6.4	7.2	10.4	11.1	12.0	11.8
C18:3 ^Δ9c,12c,15c^ (%)	0.7	0.7	0.6	0.6	0.6	0.7	0.7	0.7	0.7

Legend for fatty acids—m:n Δx, m = number of carbon atoms, *n* = number of double bonds, x = position of double bonds. ∆ECN42: difference between theoretical and experimental ECN42 content.

**Table 3:** Effect of the refining steps on the total tocopherol content (mg/kg oil).

Samples	Total Tocopherols				
	Crude oil	Degummed	Neutralized	Bleached	Deodorized
**1**	226 ± 4 ^bc^	225 ± 2 ^bc^	233 ± 5 ^c^	214 ± 4 ^a^	218 ± 2 ^ab^
**2**	170 ± 8 ^c^	152 ± 7 ^b^	138 ± 4 ^a^	142 ± 3 ^a^	157 ± 2 ^b^
**3**	154 ± 3 ^ab^	163 ± 2 ^bc^	169 ± 5 ^c^	149 ± 3 ^a^	167 ± 2 ^c^
**4**	188 ± 1 ^b^	171 ± 5 ^a^	188 ± 4 ^b^	216 ± 3 ^c^	181 ± 4 ^ab^
**5**	142 ± 2 ^b^	141 ± 4 ^b^	145 ± 3 ^b^	126 ± 5 ^a^	141 ± 3 ^b^
**6**	229 ± 2 ^a^	229 ± 5 ^a^	228 ± 2 ^a^	232 ± 3 ^a^	229 ± 2 ^a^
**7**	296 ± 3 ^b^	315 ±12 ^bc^	310 ± 3 ^b^	328 ± 6 ^c^	271 ± 5 ^a^
**8**	324 ± 6 ^b^	338 ± 5 ^b^	333 ± 2 ^b^	330 ± 5 ^b^	288 ±18 ^a^
**9**	344 ± 5 ^c^	346 ± 3 ^c^	335 ±12 ^bc^	309 ±11 ^ab^	287 ±13 ^a^

Results are expressed as the mean ± standard deviation (*n* = 3). Total tocopherol content was calculated as the sum of α-, γ-, and δ-tocopherols. Values in the same row with different superscript letters are significantly different (*p ≤* 0.05).

**Table 4:** Oil loss in the refining process.

Quality Parameters	Samples								
	1	2	3	4	5	6	7	8	9
Free acidity (%)	4.00	5.02	4.00	4.70	5.10	3.73	2.22	2.21	2.68
Oil loss/chemical refining (%)—neutralization up to 0.2% of FFAs	3.80	4.82	3.80	4.50	4.90	3.53	2.01	2.02	2.48
Oil loss/physical refining (%)—physical flash neutralization up to 0.02% of FFAs	3.78	4.80	3.78	4.48	4.88	3.51	2.00	1.99	2.46

**Table 5:** Total tocopherol content (mg/kg oil) of commercial olive oil samples.

	Total Tocopherols
Samples	
**1**	252 ± 7
**2**	481 ± 5
**3**	388 ± 5
**4**	272 ± 9
**5**	353 ± 11
**6**	174 ± 3
**7**	394 ± 4
**8**	207 ± 8
**9**	301 ± 13
**10**	108 ± 9
**11**	199 ± 5

Total tocopherol content was calculated as the sum of α-, γ-, and δ-tocopherols.
